# Massive chest wall bleeding 3 days after lung wedge resection caused by a protruding staple

**DOI:** 10.1093/jscr/rjae620

**Published:** 2024-10-13

**Authors:** Jincheng Fang, Quan Liu, Chuangyan Wu, Jinsong Li

**Affiliations:** Department of Thoracic Surgery, Union Hospital, Tongji Medical College, Huazhong University of Science and Technology, 1277 Jiefang Avenue, Wuhan, 430022, Hubei, China; Department of Thoracic Surgery, Union Hospital, Tongji Medical College, Huazhong University of Science and Technology, 1277 Jiefang Avenue, Wuhan, 430022, Hubei, China; Department of Thoracic Surgery, Union Hospital, Tongji Medical College, Huazhong University of Science and Technology, 1277 Jiefang Avenue, Wuhan, 430022, Hubei, China; Department of Thoracic Surgery, Union Hospital, Tongji Medical College, Huazhong University of Science and Technology, 1277 Jiefang Avenue, Wuhan, 430022, Hubei, China

**Keywords:** postoperative bleeding, hemothorax, endostapler, lung wedge resection

## Abstract

We report a case of massive chest wall bleeding after lung wedge resection caused by a protruding staple. On the third postoperative day, the patient experienced sudden left posterior back pain without any apparent trigger, accompanied by signs of shock. Computed tomography imaging revealed a significant accumulation of blood in the pleural cavity on the side of the surgery. A reoperation was performed, during which we identified active arterial bleeding from a small vessel at the second intercostal space on the posterior chest wall. Hemostasis was achieved using electrocautery. Further examination revealed a protruding staple at the left upper lobe resection margin, which we speculated was likely causing abrasion against the chest wall and leading to the bleeding. This case reveals the potential risk posed by protruding staples. Appropriate precautions should be taken to prevent this rare but dangerous occurrence.

## Introduction

Postoperative bleeding following thoracic surgery is a highly dangerous complication that often requires emergency reoperation and can be life-threatening. While the widespread use of disposable staplers has effectively reduced the incidence of postoperative bleeding, the staplers themselves can occasionally cause such bleeding [1]. However, these cases are extremely rare. We herein report an unusual case of postoperative hemothorax caused by a protruding staple at the resection margin 3 days after a lung wedge resection. No evident triggers were observed before the bleeding began, complicating the diagnosis and management of this urgent condition.

## Case presentation

**Figure 1:**
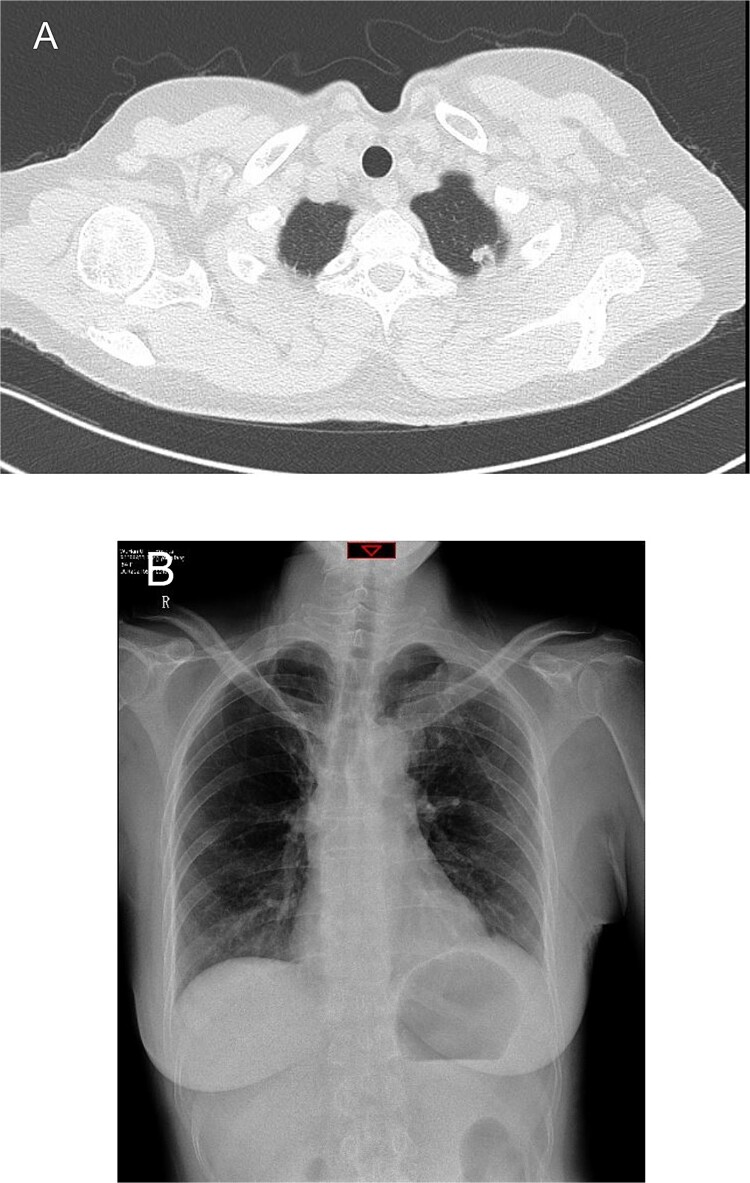
Preoperative chest computed tomography and postoperative day one chest X-ray. (A) Computed tomography shows a solid nodule in the S1+2 segment of the left upper lobe measuring 11 mm × 8 mm. (B) Postoperative day one chest X-ray indicates routine postoperative changes.

A 54-year-old female presented with a solid nodule measuring 11 mm × 8 mm in the S1+2 segment of the left upper lobe, detected on preoperative computed tomography (CT) (Fig. 1A). She had a history of right upper lobe segmentectomy performed 2 years prior, with pathology revealing atypical hyperplasia. We planned to proceed with a uniportal VATS left upper lobe wedge resection.

During surgery, a 4 cm incision was made at the fifth intercostal space. No evident pleural adhesions were found. The nodule was palpated at the posterior apex of the left upper lobe. Resection was performed using an endoscopic linear stapler with three blue cartridges and one green cartridge (Hitcmmed, Ningbo, China). Mediastinal lymph nodes from groups 5, 6, 7, and 10 were sampled. The procedure was uneventful.

On the first postoperative day, the patient’s vital signs were stable, with ~200 ml of serosanguineous fluid drained from the chest tube. Chest X-rays showed normal findings on the day after the operation (Fig. 1B). The patient was mobilized on the second day, with chest tube drainage decreasing to 100 ml, allowing for chest tube removal.

**Figure 2:**
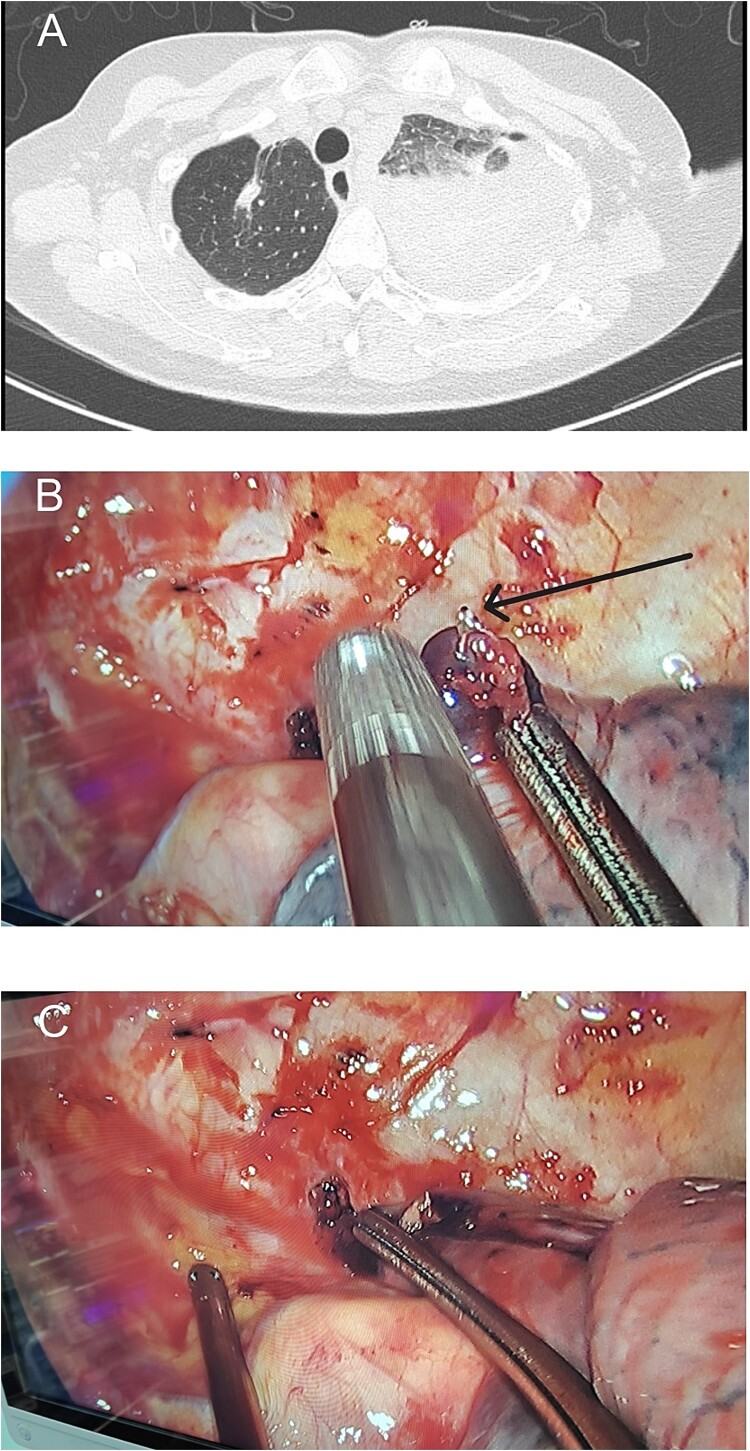
Preoperative chest computed tomography and intraoperative findings on postoperative day 3. (A) Computed tomography shows a large amount of fluid in the left pleural cavity, indicative of postoperative hemorrhage. (B) A protruding staple (black arrow) is observed at the resection margin of the left upper lung, near the site of chest wall bleeding. (C) During surgery, we attempted to reproduce the position that caused the bleeding.

On the third postoperative day, the patient experienced sudden, severe left shoulder and back pain while semi-recumbent. She appeared distressed, pale, and diaphoretic, with a blood pressure of 79/51 mmHg and a heart rate of 116 bpm. Rapid fluid resuscitation and dopamine infusion were initiated. An emergent chest CT revealed a large hemothorax, suggesting postoperative bleeding (Fig. 2A).

**Figure 3:**
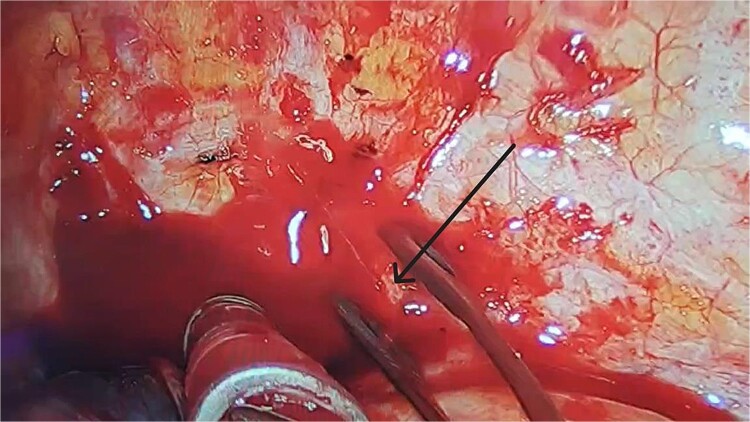
After clearing the blood clots, we observed a small artery on the surface of the posterior chest wall, around the second intercostal space, that was continuously bleeding in a jet-like manner (black arrow).

Immediate reoperation was performed. Upon re-entering the chest through the original incision, a substantial amount of blood clots were found. Active arterial bleeding was observed from a small vessel at the second intercostal space on the posterior chest wall, presuming to be the left second intercostal artery (Fig. 3). Hemostasis was achieved using electrocautery. Additionally, a protruding staple at the left upper lobe resection margin was noted, likely causing abrasion against the chest wall (Fig. 2B and C). The staple was removed, then the lung tissue was sutured using 4-0 non-absorbable monofilament. Hemostatic agents were applied to the bleeding sites. The total blood loss, including intraoperative and chest cavity blood, was ~2800 ml.

Postoperative recovery was smooth, with chest X-rays on the fourth day showing full lung re-expansion and no significant effusion. The patient was discharged on the sixth postoperative day.

## Discussion

Postoperative hemothorax is a common complication in thoracic surgery, potentially leading to severe outcomes and even being life-threatening, often necessitating urgent reoperation to control the bleeding. The introduction of thoracoscopic surgery with the use of disposable linear cutting staplers has significantly reduced the incidence of postoperative bleeding in recent years. These staplers not only lower the risk of postoperative bleeding but also significantly decrease the incidence of prolonged air leaks [1].

However, the use of staplers is not without risks. There have been reported cases where staplers have caused postoperative hemothorax. For instance, Qu *et al.* [2] reviewed cases of progressive chest wall bleeding caused by bronchial stump nails in patients who underwent lobectomy. Out of ~5000 lobectomies, four patients required reexploration due to bleeding from bronchial stump nails. Similarly, Kanai *et al.* [3] described a lethal hemothorax following a thoracoscopic lung biopsy using an endostapler with bioabsorbable tissue reinforcement, attributing the bleeding to the reinforced material on the surgical stump scratching the chest wall. Negishi *et al.* [4] reported a life-threatening hemothorax occurring 40 days after a pulmonary segmentectomy, caused by active bleeding from an intercostal artery near the staple line, likely due to the staple line scratching the chest wall. In patients with one-lung ventilation, the bronchial stump typically does not contact the pleura. However, when patients sit up and cough early in the morning, there is a risk of the nails hooking onto the pleura, leading to active bleeding. Additionally, postoperative hemothorax can also be caused by sutures. Kawamura *et al.* [5] reported a rare case of delayed postoperative bleeding caused by the sharp tip of a suture. The patient developed a hemothorax 11 days after a right lower lobectomy for lung cancer, with emergency thoracotomy revealing arterial bleeding from a pinhole injury in the parietal pleura caused by a non-absorbable suture tip used during the initial surgery.

In our case, the patient suddenly developed pain and signs of shock on the third postoperative day, without any apparent trigger such as coughing or movement. This sudden onset and rapid progression underscored the need for prompt detection and emergency reoperation to prevent irreversible severe consequences.

## Conclusion

To prevent such occurrences, surgeons should be aware of the potential risks posed by staples and sutures in causing postoperative hemothorax. Precautions should include trimming the sharp edges of staple lines or unstable staples and cutting suture threads to appropriate lengths to avoid creating sharp tips. These measures are essential to mitigate the risk of postoperative bleeding and ensure patient safety.

## Data Availability

Not applicable.
